# Experimental study on the durability of the polydopamine functionalized gas–liquid–solid microreactor for nitrobenzene hydrogenation

**DOI:** 10.1039/c7ra12460k

**Published:** 2018-02-02

**Authors:** Xun Zhu, Hao Feng, Rong Chen, Qiang Liao, Dingding Ye, Biao Zhang, Jian Liu, Ming Liu, Gang Chen

**Affiliations:** Key Laboratory of Low-Grade Energy Utilization Technologies and Systems (Chongqing University), Ministry of Education Chongqing 400030 China zhuxun@cqu.edu.cn +86-23-65102474 +86-23-65102474; Institute of Engineering Thermophysics, Chongqing University Chongqing 400030 China

## Abstract

As a promising technique for multiphase catalytic reactions, the widespread applications of gas–liquid–solid microreactors are still limited by poor durability. Hence, in this work, a method for the preparation of Pd nanocatalysts inside a gas–liquid–solid microreactor was proposed to realize long-term durability using electroless deposition on the polydopamine functionalized surface followed by hydrogen reduction. This method not only increases the utilization efficiency of the Pd ions but also improves the durability of the microreactor. The chemical composition and topography characterization of the fabricated catalyst layer were tested using XPS and FESEM, respectively. The results indicated that the incorporation of hydrogen reduction resulted in nearly all palladium ions being reduced and the palladium nanoparticles were dispersed uniformly on the polydopamine modified surface. The microreactor prepared by this method exhibited high durability and high nitrobenzene conversion as compared to the traditional electroless catalyst deposition. Besides, it was shown that the increased inlet nitrobenzene concentration and flow rates played a negative role in the durability. The longer microreactor exhibited a better durability.

## Abstract 2

As a promising technique for multiphase catalytic reactions, the widespread applications of gas–liquid–solid microreactors are still limited by poor durability.

## Graphical abstract

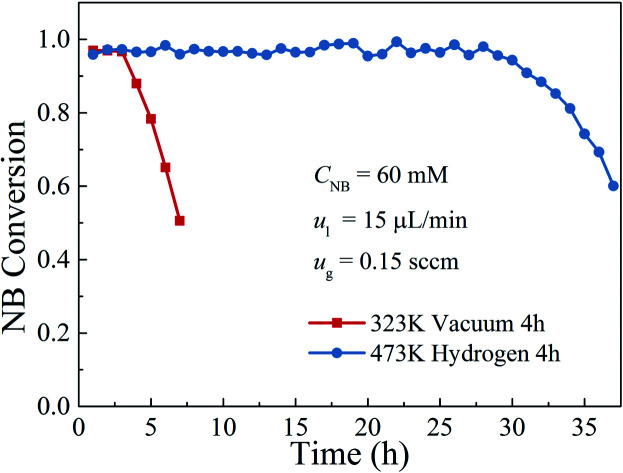

## 1. Introduction

Multiphase catalytic reactions, such as catalytic cracking, catalytic reforming, catalytic hydrogenation, *etc.*, have been widely used in the chemical and pharmaceutical industries.^1–4^ In these applications, to reduce the potential mass transfer resistance between the phases and improve the reaction rate, the pressure or/and temperature are usually increased, which will increase not only the explosion potential but also the processing and manufacturing costs.^5^ Microreactors have an intrinsically large interfacial area per unit volume and have proven to be able to dramatically enhance heat and mass transfer as well as reduce the explosion potential, making them suitable for multiphase catalytic reactions.^6–11^

To date, numerous studies have been carried out to investigate the microreactor technologies for various catalytic reactions, such as the catalytic oxidation,^12^ the catalytic hydrogenation,^6^ the catalytic combustion,^13^*etc.* It has been found that the property of the prepared catalyst layer inside the microreactor is crucial to affect the microreactor performance. Therefore, a variety of attempts have been made to prepare the catalyst layer.^14,15^ Metal nanoparticles that exhibit high catalytic activity, such as nickel, copper, palladium, platinum, *etc.*, have been chosen as the catalysts towards various catalytic reactions by sputtering, impregnation and sol–gel methods, *etc.*^16–18^ Generally, catalysts prepared by these methods could guarantee high activity. However, the calcination process during the fabrication is typically required to improve the adhesion with the substrate, which not only increases the manufacturing cost and potential risk but also limits the selection of materials. In addition to the catalytic activity, the durability of the fabricated catalyst layer is another key issue, which directly affects the industrial applications of the microreactor technologies. Unfortunately, the durability of the existing catalyst layers inside the microreactor fabricated by the conventional methods is still poor.^17,18^ Therefore, facile methods are needed for preparing catalyst layer with good durability and activity in the microreactors.

To address the above-mentioned issues, extensive efforts have been devoted to this area. For example, Kobayashi *et al.*^6^ immobilized the polymer encapsulated Pd nanoparticles inside the glass-fabricated microreactor. Hornung *et al.*^19–21^ deposited the metal nanoparticles on the polyelectrolyte using layer by layer self-assembly technology. Although significant progress has been made, there still exist problems of relatively low activity or substrate selectivity to the catalyst. Previous studies^22,23^ have indicated that adhesive proteins secreted by mussels are able to attach onto virtually various substrates, including organic and inorganic materials. Inspired by the adhesive proteins in mussels, Lee *et al.*^24–26^ used the self-polymerization of dopamine to form thin polydopamine film on various substrates, and demonstrated that the fabricated polydopamine film was excellent for the electroless deposition, which provided a new, facile method for nanocatalysts deposition on both conductive and insulated substrates. After that, successful immobilizations of metal nanoparticles on the functionalized polydopamine surface have been reported.^27–29^ However, the electroless deposition of metal nanoparticles on the functionalized polydopamine surface still faces a problem, that is, there remains a large amount of residual metal ions on the polydopamine surface as a result of the limited reduction ability of polydopamine. Incomplete reduction not only decreases the utilization efficiency of the metal ions but also results in fast deactivation of the nanocatalysts and thus reduces reactant conversion and the durability of the microreactor. To solve this problem, a gas–liquid–solid microreactor was developed with the polydopamine functionalized surface coated with highly-active palladium nanocatalysts by the electroless deposition plus using hydrogen as a reducing agent.^30^ The experimental results showed the developed gas–liquid–solid microreactor was able to yield good catalytic activity. However, as one of the key points reflecting the performance of the microreactor, the durability of the microreactor fabricated by this method remains unknown. For this reason, particular attention of this work was paid to the evaluation of the durability of the gas–liquid–solid microreactor fabricated by this method under various operation conditions. For comparison, the differences in the chemical composition and morphology as well as the performance between the catalyst layer without and with further hydrogen reduction under different operational conditions were studied.

## 2. Experimental

### 2.1 Materials and chemicals

Commercially available PTFE (polytetrafluoroethylene) tube with the inner diameter of 0.6 mm and outer diameter of 1.0 mm was used to fabricate the microreactor. Dopamine hydrochloride and Tris (tris(hydroxymethyl) aminomethane) were purchased from Aladdin Industrial Inc (Shanghai, China) and GEN-VIEW Scientific Inc (Florida, USA), respectively. Palladium chloride was obtained from Sino-Platinum Metals Co. Ltd (Kunming, China). Potassium chloride, ethanol, nitrobenzene were acquired from Chongqing Chuandong Chemical Co. Ltd (Chongqing, China). The chemicals were analytical reagent grade and used as-received. Deionized water was obtained from an Ultrapure Water System for Laboratory (ROMB, China).

### 2.2 Dopamine self-polymerization on the inner surface of PTFE tube

Alkaline dopamine aqueous solution was prepared by dissolving dopamine in 10 mM Tris buffer solution with the dopamine concentration of 2 mg mL^−1^. In order to form an adherent polydopamine layer on the inner surface of the PTFE tube, the prepared dopamine aqueous solution was firstly introduced into the PTFE tube at a flow rate of 1 mL h^−1^ for 5 h using a syringe pump (LSP04-1A, Longer-Pump, China). In the following, the PTFE tube was flushed with 10 mL deionized water at a flow rate of 0.5 mL min^−1^. Then, nitrogen gas was continuously fed into the tube at a flow rate of 2 sccm and heated at 338 K for 1 h by a mass flow controller (FMA-2616A-I, Omega, USA) and an electric heating plate (OMHP-3C, OU-mai, China), respectively. After the completion of these steps, a polydopamine film on the inner wall of the PTFE tube could be acquired. The obtained modified PTFE tube was denoted as PDA/PTFE.

### 2.3 Electroless deposition of palladium nanoparticles on PDA modified surface

**Fig. 1:**
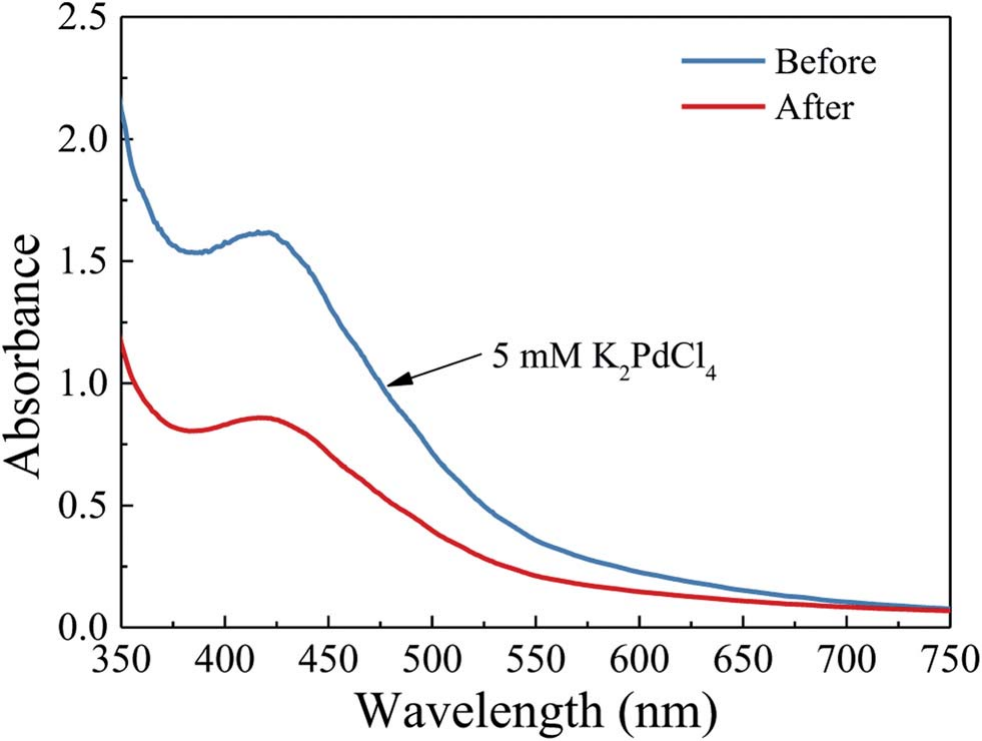
UV-vis spectra of the K_2_PdCl_4_ solution before and after the electroless deposition on the polydopamine functionalized surface.

In this stage, 5 mM K_2_PdCl_4_ aqueous solution prepared by dissolving PdCl_2_ and KCl in water (PdCl_2_: 886.65 μg mL^−1^ and KCl: 372.75 μg mL^−1^), was used as the precursor solution. The precursor solution was infiltrated into the PTFE tube with coated PDA layer at a flow rate of 50 μL min^−1^. Once the tube was full of the precursor solution, the flow rate of the precursor solution was then changed to 0.1 μL min^−1^ and maintained for 12 h to adsorb and reduce the Pd ions. After that, excess amount of water was fed into the PTFE tube several times at a flow rate of 10 μL min^−1^ to the remove of residual ions, which was followed by flushing with nitrogen gas at a flow rate of 0.1 sccm for 5–10 min to remove the remaining water. Finally, the resulting PTFE tube was placed into a vacuum tube resistance furnace (Tianjin Zhonghuan Lab Furnace Co., Ltd, China) and heated in the H_2_ environment under 473 K for 4 h to further reduce the residual Pd ions to metallic nanoparticles. The obtained sample was denoted as Pd-PDA/PTFE in the following discussion. To estimate the Pd content in the microreactor, a UV-visible spectrophotometer (T6-1650E, Persee, China) with the wavelength range from 190 nm to 1100 nm at 1 nm resolution was used to measure the difference between the 5 mM K_2_PdCl_4_ solution and the collected K_2_PdCl_4_ solution after deposition. The result is shown in Fig. 1. As can be seen, the solutions before and after the deposition both showed a peak at 416 nm in the UV-vis spectrum, which corresponded to palladium ions.^31^ The absorbance decreased from 1.62 to 0.86 after the deposition, indicating that the concentration of the palladium ions in the collected K_2_PdCl_4_ solution was about 2.65 mM. Considering that about 360 μL 5 mM K_2_PdCl_4_ solution was introduced into the microreactor for the Pd deposition, the content of Pd catalyst in the microreactor was thus estimated to be about 90 μg (4.77 μg cm^−2^).

### 2.4 Materials characterization and experimental setup

**Fig. 2:**
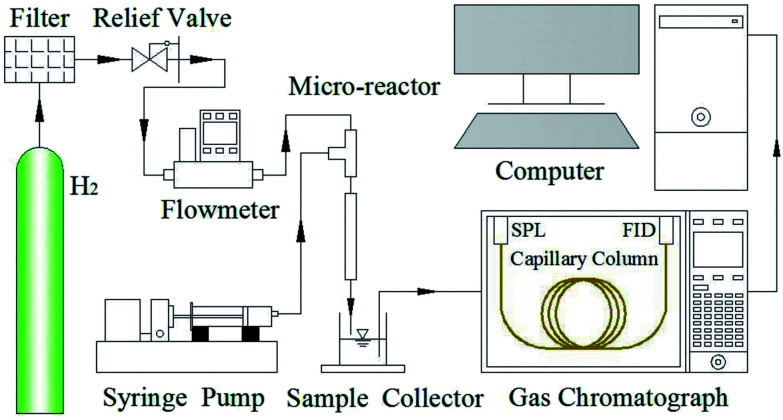
Schematic diagram of the experimental system.

The chemical compositions of original and modified PTFE, Pd-PDA/PTFE were determined by XPS using an ESCALAB 250Xi XPS system (Thermo, USA) with Al Kα radiation (*hν* = 1486.6 eV) at a reduced power of 150 W. The surface morphologies of these samples were characterized by using a field emission scanning electron microscope (S4800, Hitachi High Technologies, Japan) with the accelerating voltage of 3 kV. Fig. 2 shows the schematic diagram of the experimental system. To supply the gaseous and liquid reactants stably and continuously, the liquid reactant prepared by dissolving nitrobenzene in ethanol aqueous solution (C_2_H_5_OH : H_2_O = 7 : 3, v/v), and the gaseous reactant of high-purity hydrogen were simultaneously fed into the microreactor using a syringe pump (LSP01-1BH, Longer-Pump, China) and a mass flow controller (FMA-2602A-I, Omega, USA) through a home-made connector. The effluent was collected and measured by a gas chromatograph (GC-2010 Plus, Shimadzu, Japan) equipped with a split/splitless injection unit (SPL), a capillary column (Rtx-1301, 30 m × 0.32 mm, Restek, USA) and a flame ionization detector (FID) to measure the concentration of nitrobenzene and the desired product of aniline.

## 3. Results and discussion

### 3.1 Characterization of dopamine self-polymerization

**Fig. 3:**
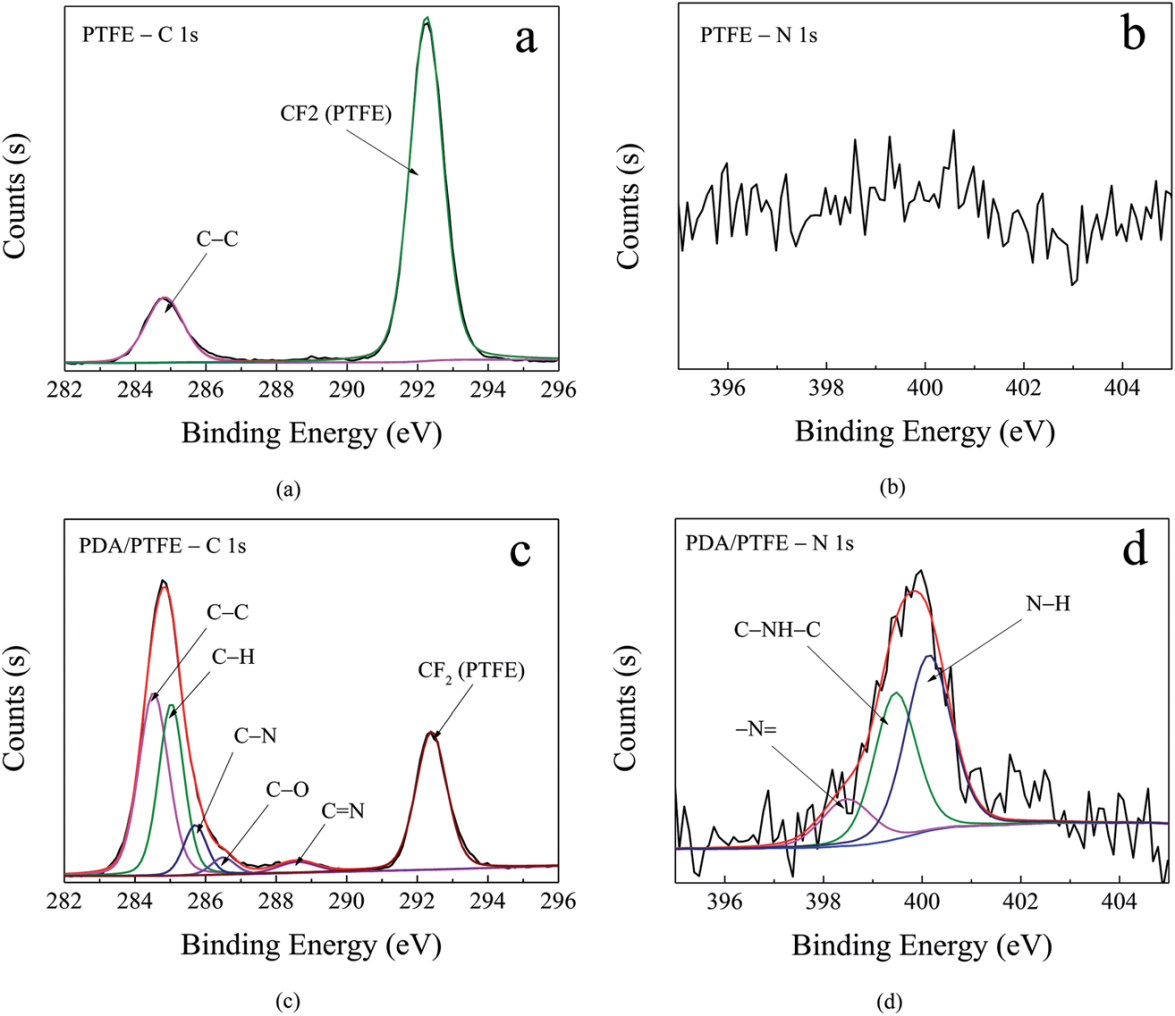
XPS C 1s and N 1s core-level spectra of PTFE (a and b) and PDA/PTFE (c and d).

To elaborate the formation of the PDA layer, the chemical compositions of the original and PDA modified PTFE were characterized by XPS. Fig. 3 shows the narrow scan spectra and curve fitting of the C 1s and N 1s for the pristine and PDA modified PTFE. As can be seen in Fig. 3a, the C 1s core-level spectrum of the pristine PTFE tube can be curve-fitted into two peaks at 284.8 eV and 292.3 eV, which represented the C–C and CF2, respectively. As compared with the pristine PTFE, four additional peaks represented the C–H (285.0 eV), C–N (285.7 eV), C–O (286.5 eV), C=N (288.6 eV), respectively, existed in the PDA modified PTFE (see Fig. 3c). The appearance of C=N in the PDA/PTFE was consistent with the structure of PDA, which indicated the existence of PDA. In order to further verify the composition of PDA on the inner wall of the PTFE tube, the N 1s spectrum was also curve-fitted. Compared with the pristine PTFE (see Fig. 3b), the N 1s of PDA/PTFE shown in Fig. 3d can be decomposed into three typical peaks assigned to –N= (398.5 eV), C–NH–C (399.5 eV) and N–H (400.1 eV). During the self-polymerization process of dopamine, the structure evolution of amine group in dopamine formed –N= and C–NH–C, and the N–H was attributed to the amine group.^32,33^ The existence of –N= and C–NH–C was in accordance with the PDA structure, which also demonstrated the existence of PDA. In conclusion, the XPS results suggest that PDA layer has been successfully adhered to the inner surface of PTFE tube through the self-polymerization of dopamine.

**Fig. 4:**
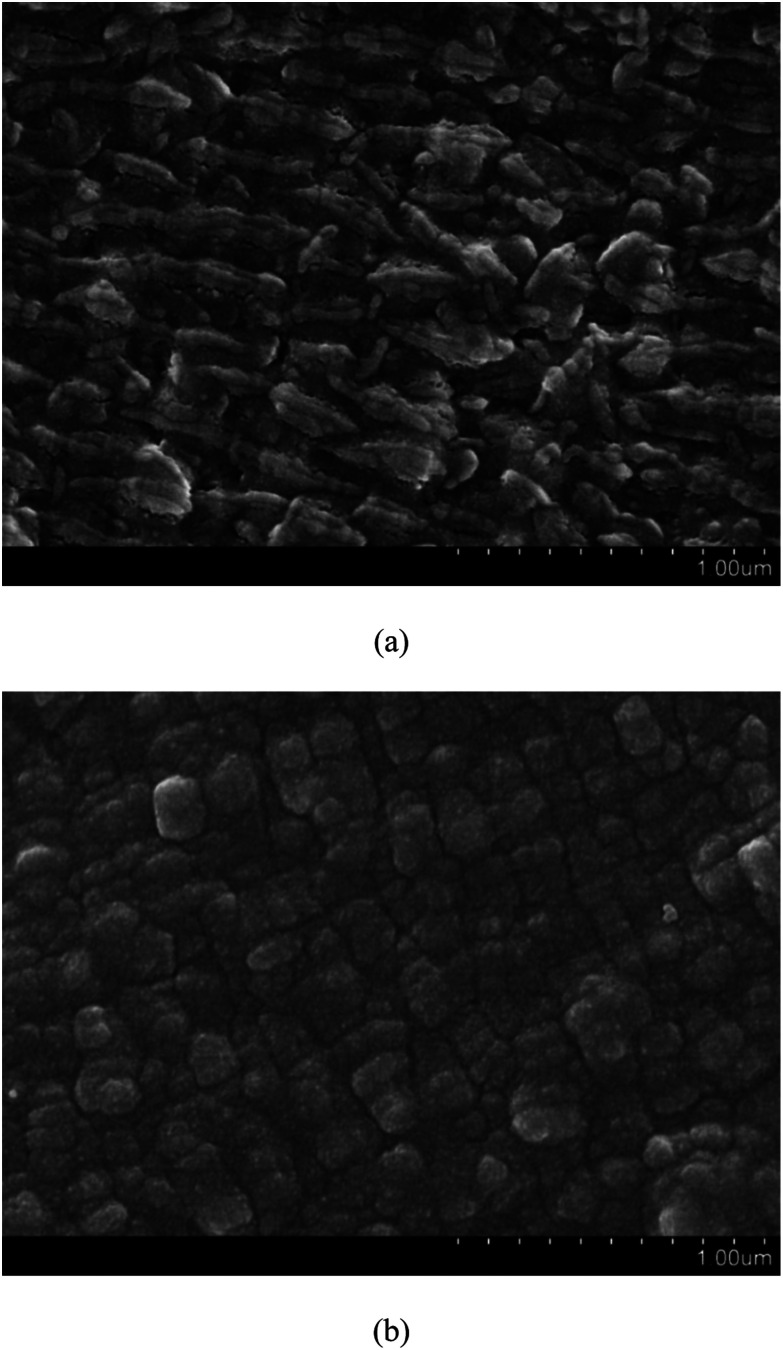
FESEM images of (a) PTFE and (b) PDA/PTFE.

Fig. 4 shows the FESEM images of pristine PTFE and PDA/PTFE at different magnifications. As can be seen, prior to the PDA formation, the PTFE tube exhibited a striped surface microstructure. After coating the PDA on the PTFE surface, a cubic surface microstructure was observed. The difference in the surface morphology between the PTFE and the PDA/PTFE indicated a uniform PDA coating was formed on the inner surface of the PTFE tube.

### 3.2 Characterization of Pd nanoparticles on PDA/PTFE surface

**Fig. 5:**
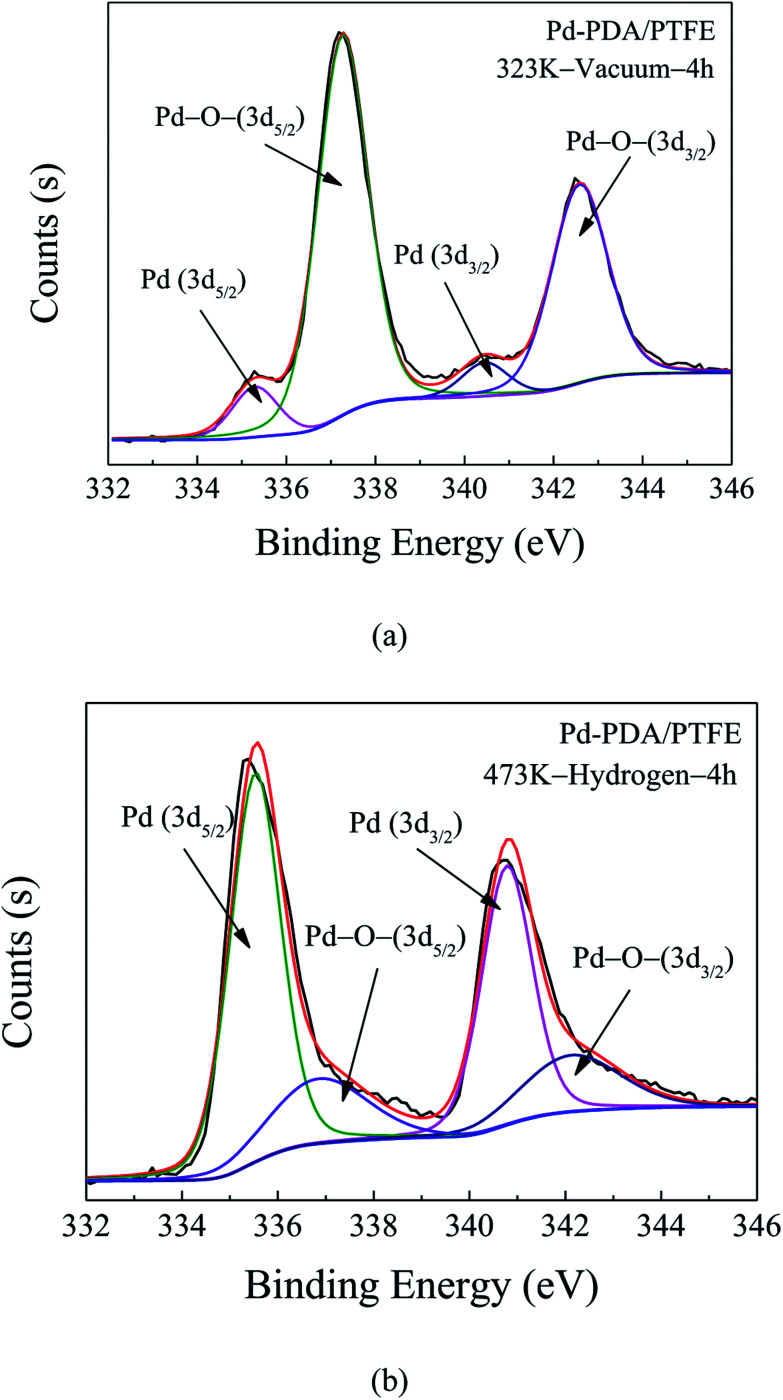
XPS Pd 3d core-level spectra of Pd-PDA/PTFE catalyst layer without (a) and with (b) hydrogen reduction.

In order to obtain the surface morphology and chemical structure of the PDA layer coated with palladium, FESEM and XPS measurements were performed. The XPS Pd 3d core-level spectrum for Pd nanoparticles only reduced by the PDA is presented in Fig. 5a. It was shown that the deconvolution of the Pd 3d core-level spectrum of the Pd-PDA layer resulted in four peaks at 335.4 eV, 337.2 eV, 340.7 eV and 342.7 eV, which were assigned to Pd^0^ (3d_5/2_), Pd–O– (3d_5/2_), Pd^0^ (3d_3/2_) and Pd–O– (3d_3/2_), respectively. The Pd^0^ component came from the reduction of Pd^2+^ to Pd^0^ since the existence of catechol and N-containing functional groups from the PDA layer, which acted not only as an adhesive but also as a reducing agent for the formation of Pd nanoparticles.^27,32^ In this deposition step, since catechol could serve as a good ligand to form chelates with palladium ions, palladium ions were able to bound with the anionic oxygen of the catechol contained in the PDA. However, due to the limited reduction ability of PDA, there always existed a large amount of Pd–O– components at the surface. As mentioned earlier, to further reduce the residual palladium ions on the PDA surface to metallic palladium nanoparticles and increase the precursor utilization efficiency, extra reducing agent of hydrogen gas was introduced in this work. Similarly, the XPS Pd 3d core-level spectrum for Pd nanoparticles after the hydrogen reduction was measured, and the result is shown in Fig. 5b. As shown, the Pd 3d spectrum always consists of Pd^0^ (3d_5/2_), Pd–O– (3d_5/2_), Pd^0^ (3d_3/2_) and Pd–O– (3d_3/2_). However, compared to the result without hydrogen reduction, the peak components associated with Pd–O– were much smaller, which indicated that most of the palladium ions have been successfully reduced and deposited on the PDA/PTFE surface in the metallic state.

**Fig. 6:**
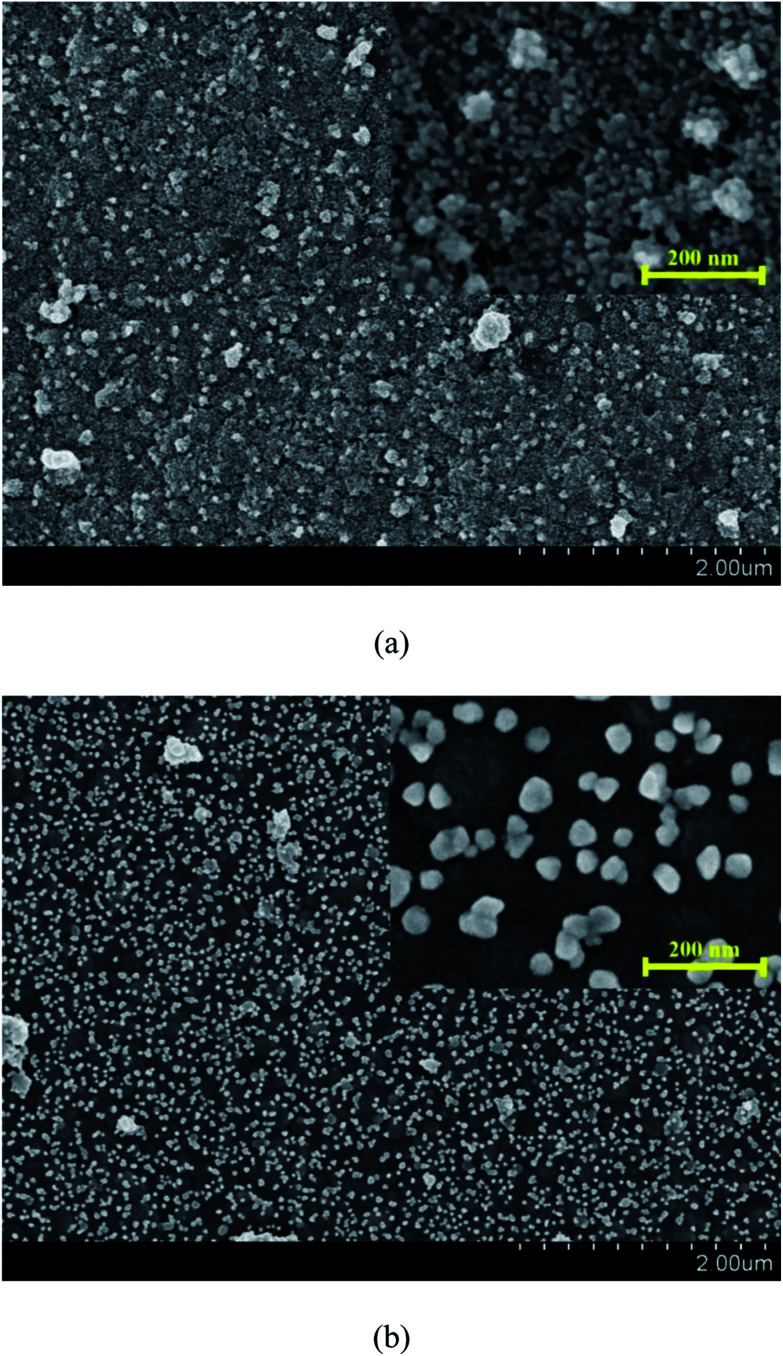
FESEM images of Pd-PDA/PTFE catalyst layer without (a) and with (b) hydrogen reduction.

Fig. 6 shows the surface morphology of the Pd-PDA surfaces without and with hydrogen reduction. As can be seen in Fig. 6a, the size of the vast majority of the Pd nanoparticles lied in the range of about 15 nm, which were dispersed uniformly on the PDA surface. Fig. 6b shows the morphology result of the catalyst with the further reduction by hydrogen. As can be seen, the discrete Pd nanoparticles were well distributed on the PDA surface, but the particles sizes were larger than those without further hydrogen reduction. Two reasons contributed to this phenomenon. First, when the Pd ions adsorbed on the PDA surface uniformly, although they could be reduced by the PDA, the reduction ability of the PDA was weak. Hence, only a small part of the adsorbed Pd ions could be reduced to metallic state and the majority was still kept at ion state.^32^ In this case, the Pd nanoparticles were unable to exhibit large particle size. On the other hand, for the case with hydrogen reduction, nearly all adsorbed Pd ions could be reduced so that the Pd nanoparticles could grow bigger during the reduction process. Moreover, a high operation temperature of 473 K was required in this process. Such a high reduction temperature may lead to easy agglomeration of Pd nanoparticles to form larger particles.^34^ As a consequence, although almost all the Pd ions were reduced to elemental palladium with the further hydrogen reduction, the particle size was also increased.

### 3.3 Evaluation of durability

**Fig. 7:**
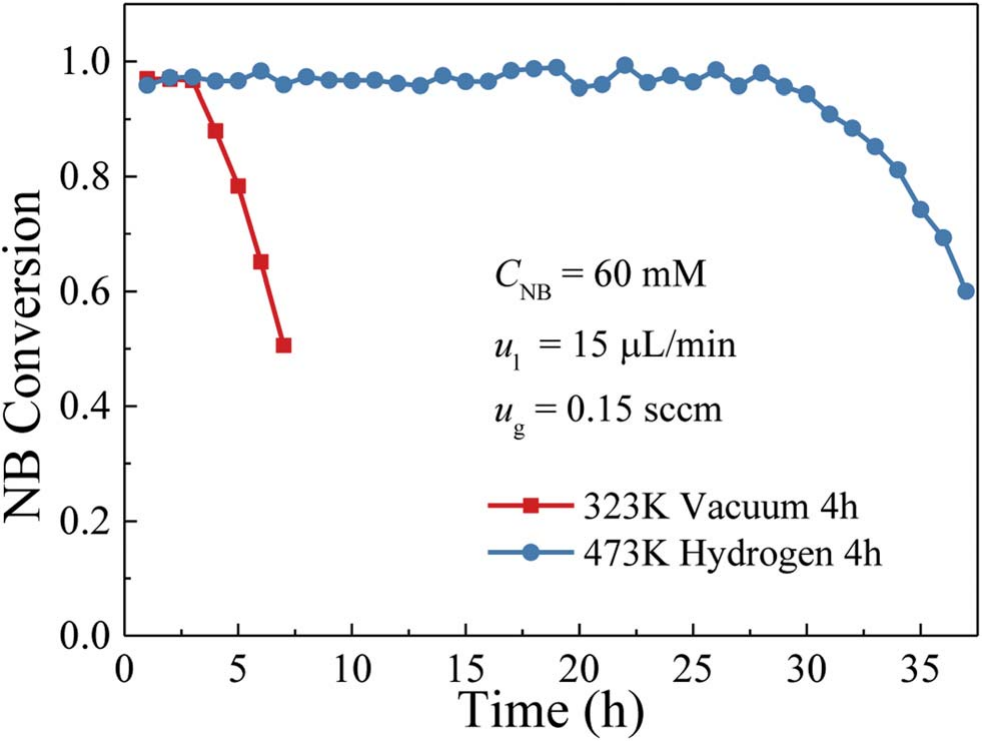
Variations of the nitrobenzene conversion with the reaction time between the catalyst layer with and without hydrogen reduction.

Durability, as one of the important indicators to evaluate the performance of the catalyst layer in the microreactor, plays a critical role in the industrial applications. Thus, a long-term operation was carried out for the durability characterizations of the catalyst layers with and without further hydrogen reduction. In this test, the inlet nitrobenzene concentration was kept at 60 mM, hydrogen and nitrobenzene flow rates were set at 0.15 sccm and 15 μL min^−1^, respectively. The variations of the nitrobenzene conversion with the operation time are shown in Fig. 7. As can be seen, for the catalyst layer without hydrogen reduction, during the first 3 h operation, the average nitrobenzene conversion could reach over 97%, but it was decreased to 50.5% with further increasing the operation time. However, for the catalyst layer with further reduction by hydrogen, over the entire operation period of 37 h, the nitrobenzene conversion could be kept stable during the first 28 h operation with an average nitrobenzene conversion of about 97% and then declined gradually to 60.03% in the next 9 h operation. These results indicated that the catalyst layer prepared with hydrogen reduction could not only perform an excellent catalytic activity for the hydrogenation of nitrobenzene but also showed much better durability than did the one without hydrogen reduction. Such a big gap in the catalyst durability may be attributed to the differences in the proportions of Pd metallic state. It is well known that the nitrobenzene hydrogenation is a structure insensitive catalytic reaction. Its reaction rate does not depend on the crystal surface structure of the active metal, but only relies on the amount of metallic Pd catalyst exposed to the reactants.^35^ For the catalyst layer without hydrogen reduction, a significant amount of Pd ions has not been completely reduced so that the Pd–O– species existed in the catalyst layer were unable to catalyze the nitrobenzene hydrogenation. However, there was still a small amount of the reduced Pd, which had good activity to the nitrobenzene hydrogenation. In this case, the catalyst layer with hydrogen reduction could exhibit good performance at the initial period of 3 hours. However, during the reaction process, the catalyst deactivation as a result of the catalyst poison, nanocatalysts agglomeration, *etc.*, is unavoidable. The generated intermediates/by-products (such as nitrosobenzene, azoxybenzene) have a strong adsorption on the active sites of the Pd nanocatalysts and can directly poison the Pd nanocatalyst.^36,37^ As a result, for the catalyst layer without hydrogen reduction, although good performance could be obtained at the beginning, the durability was rather poor. However, for the catalyst layer with hydrogen reduction, almost all the Pd ions had been reduced and utilized, the Pd species exposed to the reactants was much more than those without hydrogen reduction. Besides, the Pd particle size was large, making it a little difficult to agglomerate during the operation. Hence, it could perform a much better durability for the nitrobenzene hydrogenation compared with the catalyst layer without hydrogen reduction. In addition, we also compared the catalytic performance and durability of the catalysts fabricated in this study with those conventional methods and the results are listed in Table 1. As shown, in comparison to the catalyst layer in the conventional microreactors and the catalyst in the batch reactor, the catalysts fabricated by the proposed method in this work exhibited not only high nitrobenzene conversion but also relatively good durability. It is further demonstrated that the method proposed in this study shows the promising potential for future industrial applications.

**Table 1:** Comparison of the catalytic performance and durability^a^

Catalyst	Reactor	*C*_NB_/mM	*t*/s	*X*_NB_	Durability/h	Ref.
Pd film	Micro	400	17	27%	1	16
Pt/TiO_2_	Micro	50	12	93%	8	17
Pd/Al_2_O_3_	Micro	30	400	90%	10	18
Pt/CNT	Batch	98	2400	90%	—	35
Pd/PDA	Micro	60	112	97%	28	This work

a *C*_NB_ – initial/inlet nitrobenzene concentration, *X*_NB_ – nitrobenzene conversion, *t* – residence/reaction time.

### 3.4 Effect of the operating parameters on durability

To further evaluate the durability of the fabricated catalyst layer, the effects of the inlet nitrobenzene concentration, the flow rate as well as the length of the microreactor were also investigated in this work.

#### 3.4.1 Inlet nitrobenzene concentration

**Fig. 8:**
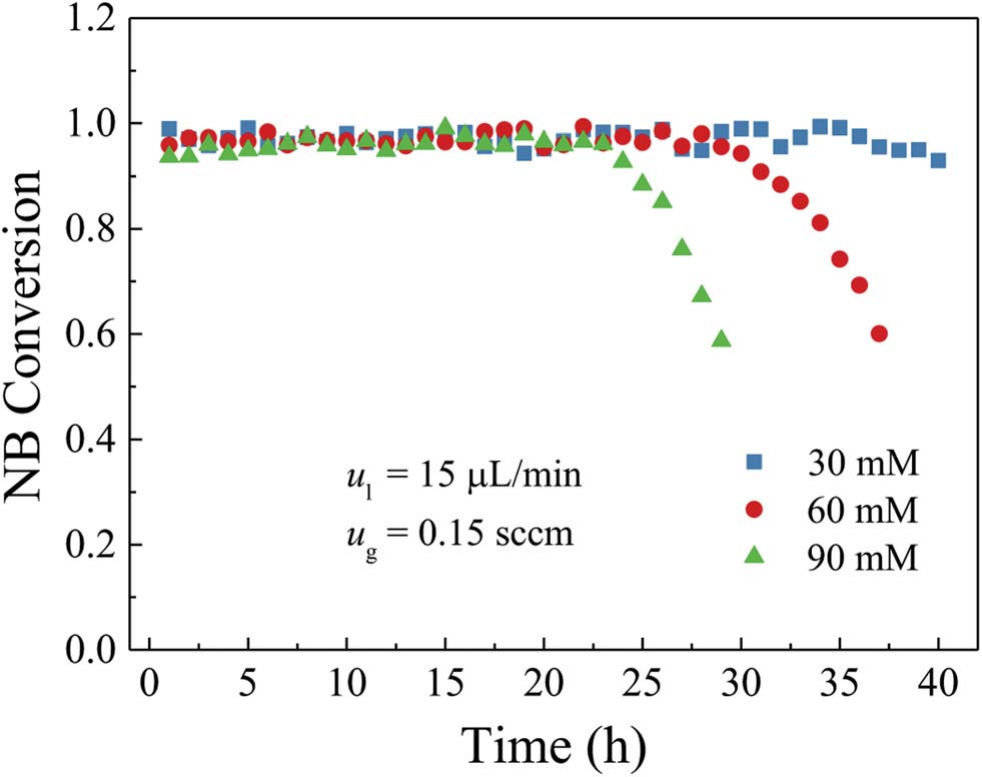
Effect of the inlet nitrobenzene concentration on the NB conversion and durability.

In this section, the inlet nitrobenzene concentration ranged from 30 mM to 90 mM. The flow rates of the gas and liquid reactants were kept at 0.15 sccm and 15 μL min^−1^, respectively. The length of the microreactor was 1.0 m. Note that the supplied hydrogen under this flow rate can ensure the supplied nitrobenzene to be completely converted. Fig. 8 shows the effect of the inlet nitrobenzene concentration on the nitrobenzene conversion over the entire operation period of 40 h. As can be seen, at the 30 mM operation, over the entire operation period, the nitrobenzene conversion was kept stable without obvious decrease and the average nitrobenzene conversion was about 97%. With the inlet nitrobenzene concentration increased to 60 mM, the nitrobenzene conversion was kept stable in the first 28 h with an average nitrobenzene conversion of about 97% and then gradually declined to about 60% in the next 9 h. And when the inlet concentration was further increased to 90 mM, the nitrobenzene conversion was rather stable in the first 23 h and then gradually declined as the operation time went on. It is clear that high nitrobenzene conversion could be obtained during the stable stage for each inlet nitrobenzene concentration, while the durability of the microreactor was decreased with increasing the inlet nitrobenzene concentration. As discussed above, nearly all Pd ions adsorbed on the PDA surface had been reduced and a large amount of Pd nanoparticles was uniformly dispersed on the surface (see Fig. 5 and 6), which provided a large number of catalytically active sites for the nitrobenzene hydrogenation. Thus, high conversion in the first stage could be obtained even at the nitrobenzene concentration of 90 mM. However, with the increase of the inlet nitrobenzene concentration, more intermediates and by-products were generated during the catalytic reaction process, which would not only cover the active sites of the catalysts but also aggravate the agglomeration of the Pd nanocatalysts.^38^ It is indicated that the decrease of the active sites exposed to the reactants hindered the catalytic reaction. Under such a circumstance, less and less nitrobenzene could be converted to aniline. The nitrobenzene conversion was lower and lower and eventually the catalytic reaction stopped. As a result, the durability of the catalyst layer inside the microreactor decreased with the increase of the inlet nitrobenzene concentration.

#### 3.4.2 Flow rate

**Fig. 9:**
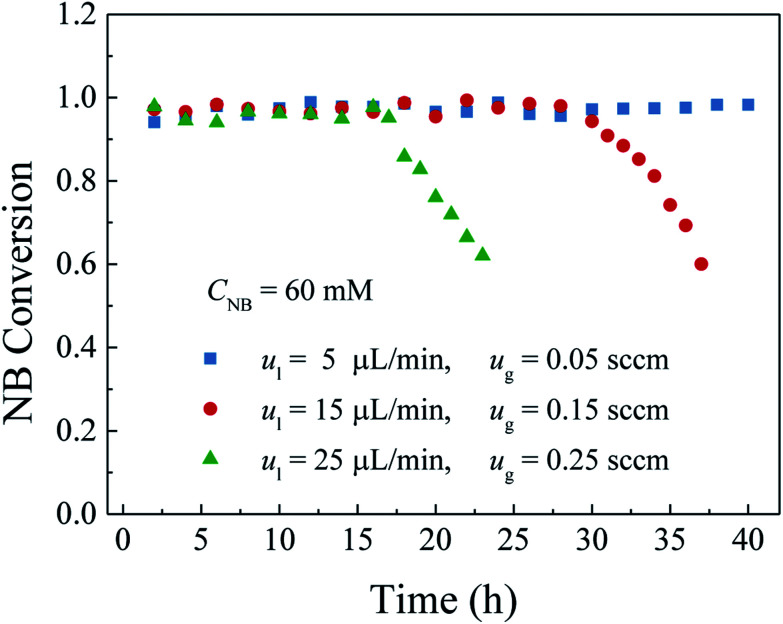
Effect of the flow rates on the NB conversion and durability.

Variations in both the liquid and gas flow rates can cause the changes of both the residence time and the load of the microreactor and thus affect the microreactor performance. In this section, therefore, the effect of the flow rates was investigated. In this experiment, the inlet nitrobenzene concentration of 60 mM and the microreactor length of 1.0 m were selected. To ensure the analogical gas–liquid two-phase flow pattern in the microreactor and sufficient supply of hydrogen for complete conversion, the flow rate ratio of gas to liquid was kept at 10. The liquid flow rates ranged from 5 μL min^−1^ to 25 μL min^−1^, with the corresponding gas flow rate ranging from 0.05 sccm, to 0.25 sccm, respectively. Because of the difference between the gas and liquid flow rates, the residence time of the reactants in the microreactor was defined as,Residence time = channel volume/gas flow ratewhere the channel volume was about 280 μL as given. Based on this definition, the residence times corresponding to the flow rates were determined to be about 336 s, 112 s and 67.2 s, respectively. Large residence time corresponds to a low flow rate. Fig. 9 shows the variation in the nitrobenzene conversion with the flow rates. Obviously, at low flow rate corresponding to the residence time of 336 s, the nitrobenzene conversion was as high as 97% in the entire operation period. When the gas and liquid flow rate were set at 15 μL min^−1^ and 0.15 sccm, which corresponded to the residence time of 112 s, the nitrobenzene conversion was stable in the first 28 h and then gradually declined. With the further increase of the flow rates, the period with stable and high nitrobenzene conversion was further decreased. The difference in the nitrobenzene conversion between the flow rates may be attributed to two aspects. On one hand, the increased flow rate means that more reactants participated the reaction and more intermediates and by-products were generated, which intensified both the adsorption of the intermediates and by-products on the active sites. Thereby, the durability of the microreactor was lowered. On the other hand, the low flow rate means longer residence time in the microreactor, which not only allowed the reactants effectively to contact with the catalyst but also lowered the load to the microreactor system. Thus, although the active sites were adsorbed by those intermediates and by-products, the overall supply of the reactants was small so that the overall amount of generated intermediates and by-products was reduced. Therefore, a stable and high nitrobenzene conversion could still be acquired, indicated the commendable durability of the microreactor. Attributed to these two reasons, low flow rate can perform a high durability for the given nitrobenzene concentration and microreactor length. The durability is decreased with the increase of the flow rates.

#### 3.4.3 Microreactor length

**Fig. 10:**
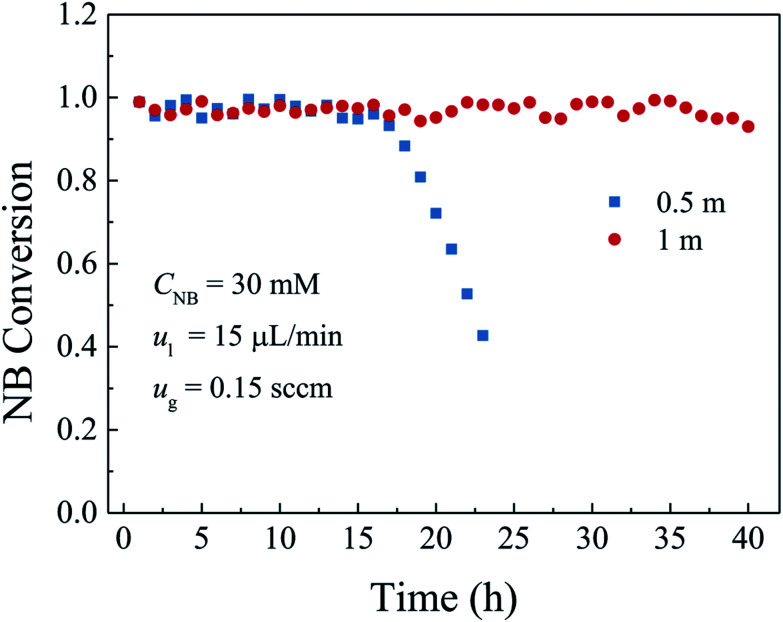
Influence of the microreactor length on the NB conversion and durability.

The length of the microreactor is another important factor affecting the durability of the microreactor because of the difference in the catalyst loading, active surface area and the residence time. To study the effect of the microreactor length, in this section, the inlet nitrobenzene concentration was kept at 30 mM, the gas and liquid flow rates were set at 0.15 sccm and 15 μL min^−1^, respectively. Two microreactor lengths of 0.5 m and 1.0 m were chosen. The experimental results are shown in Fig. 10, which indicated that the durability of the microreactor with large length was better than that with short length. This is because increasing the length of the microreactor increased the total catalyst loading and the active sites in the whole microreactor. Although the catalyst deactivation was unavoidable, excess catalyst could provide enough active sites for the promotion of the durability. On the other hand, the increased length of the microreactor increased the residence times of both the gas and liquid reactants in the microreactor, which also contributed to high nitrobenzene conversion even after a long-term operation.

## 4. Conclusions

A catalyst layer with high durability is of importance before the widespread applications of the gas–liquid–solid microreactors. In this study, a polydopamine functionalized gas–liquid–solid microreactor with the absorbed Pd ions further reduced by hydrogen to enhance the durability. The surface chemical composition of the catalyst layer fabricated by this method showed that the proportion of metallic Pd in catalyst layer reduced with hydrogen was much larger than that without additional hydrogen reduction. The morphology characterization indicated that Pd nanoparticles were well deposited and uniformly dispersed on the PDA modified surface with an average particles size of about 50 nm. The durability of the developed microreactor was evaluated by the nitrobenzene hydrogenation. It was shown that both high nitrobenzene conversion and durability were obtained for the developed microreactor. Besides, the effects of the inlet nitrobenzene concentration and the flow rates and the microreactor length on the durability were also investigated. The results showed that, for the given flow rates and microreactor length, the nitrobenzene conversion was kept stable without obvious decrease at the inlet nitrobenzene concentration of 30 mM over the entire operation period because of less adsorption of the intermediates and by-products on the active sites. When the nitrobenzene concentration was 60 mM, the nitrobenzene conversion was kept stable in the first 28 h and then gradually declined to 60% in the next 9 h. As the inlet nitrobenzene concentration was further increased to 90 mM, the durability of the microreactor was further decreased. As for the effect of the flow rates, a tendency that the durability of the microreactor was decreased with the increase of the flow rates could be obtained as a result of intensified adsorption of the intermediates and by-products on the active sites as well as the decreased residence time. Regarding the effect of the microreactor length, it was found that the durability of the microreactor increased with the microreactor length because of increased catalyst loading and residence time. The obtained results reveal that the developed gas–liquid–solid microreactor can not only yield high conversion but also promote the durability.

## Conflicts of interest

There are no conflicts to declare.

## Supplementary Material
